# Whole-exome sequencing of long-term, never relapse exceptional responders of trastuzumab-treated HER2+ metastatic breast cancer

**DOI:** 10.1038/s41416-020-0999-z

**Published:** 2020-07-27

**Authors:** Naomi Walsh, Charlotte Andrieu, Peter O’Donovan, Cecily Quinn, Alanna Maguire, Simon J. Furney, Giuseppe Gullo, John Crown

**Affiliations:** 1 National Institute for Cellular Biotechnology, School of Biotechnology, Dublin City University, Glasnevin, Dublin, Ireland; 2 Genomic Oncology Research Group, Centre for Systems Medicine, Department of Physiology and Medical Physics, Royal College of Surgeons in Ireland, Dublin, Ireland; 3 St Vincent’s University Hospital, Elm Park, Dublin, Ireland

**Keywords:** Breast cancer, Personalized medicine

## Abstract

Trastuzumab has significantly improved the overall survival of patients with HER2+ metastatic breast cancer (MBC). However, outcomes can vary, with patients progressing within 1 year of treatment or exceptional cases of complete response to trastuzumab for ≥10 years. Identification of the underlying genomic aberrations of “exceptional responders (ExRs)” compared to “rapid non-responders (NRs)” increases our understanding of the mechanisms involved in MBC progression and identification of biomarkers of trastuzumab response and resistance. Whole-exome sequencing was performed on six ExRs compared to five NR. The overall fraction of genome copy number alteration (CNA) burden was higher in NR patients (*P* = 0.07), while more significantly pronounced in copy number gains (*P* = 0.03) in NR compared to ExRs. Delineation of the distribution of CNA burden across the genome identified a greater degree of CNA burden in NR within Chr8 (*P* = 0.02) and in Chr17 (*P* = 0.06) and conferred a statistically significant benefit in overall survival. Clinical trial number: NCT01722890 [ICORG 12/09].

## Background

Historically, HER2+ metastatic breast cancer (MBC) was designated as an incurable disease. The introduction of anti-HER2 therapies such as trastuzumab has markedly improved survival^1^ and extended to 56.5 months with the combination of trastuzumab plus pertuzumab plus docetaxel.^2^ We and others^3, 4^ have reported cases of long-term durable complete response to trastuzumab in HER2+ MBC. However, to date only clinical and molecular analysis of this “exceptional” cohort exists. Somatic copy number alterations (CNAs) alter a significant proportion of the cancerous genome. CNAs dominate the breast cancer genome, with somatic mutations in breast cancer genes at low frequencies and mainly characterised in driver genes by high-throughput targeted mutation profiling.^5^ However, somatic single-nucleotide variants (SNVs) and insertions/deletions in driver genes do contribute to tumour biology.^6^ It remains unclear as to the extent in which genomic CNA burden can act as a prognostic measure of predicting response to trastuzumab in long-term, never relapse exceptional responders (ExRs) compared to rapid non-responders (NRs). To investigate this hypothesis, we present the first study of whole-exome sequencing (WES) analysing the genome CNA burden of six ExRs compared with five NRs with HER2+ MBC patients treated with trastuzumab.

## Methods

A retrospective, single institution review from 2000 to 2018 identified 295 HER2+ MBC who received treatment with trastuzumab in the metastatic setting. Informed consent was obtained and approved by St. Vincent’s University Hospital Ethics Committee. HER2+ immunohistochemical status was confirmed by pathologist (C.Q.). Analysis of patients with never relapse and >3-year overall survival follow-up data identified 40 patients. A further refined analysis revealed 11 patients with a minimum overall survival of 10 years (range 10–19 years). We performed WES on 6/11 of these patients and 5 corresponding NRs (median relapse-free survival (RFS) ≤14 months) (Supplementary Table 1). DNA from tumour and adjacent normal tissue (where available) underwent WES (Agilent SureSelect Human All Exon V4) using an Illumina HiSeq (2 × 100 bp) at a mean depth of 56×. Reads were trimmed and aligned to the hg38 reference genome using BWA^7^ and duplicate reads were marked. Base recalibration was conducted with GATK.^8^ Variant calling was performed using Mutect2 from GATK (4.1.3). CNAs were identified by EXCAVATOR2.^9^ Variant statistics and tumour mutational burden (TMB) were calculated by maftools.^10–12^

Two-sample *t* test with unequal variances was used to evaluate total genome CNA burden between ExRs and NRs, and across individual chromosomes, two-sided *P* value < 0.05 was considered statistically significant. Somatic CNA burden was used to stratify patients into high and low CNA burden groups based on the median CNA burden observed across each chromosome. The binary CNA stratification groups were further assessed using Kaplan–Meier survival estimation; *P* value < 0.05 log rank was considered statistically significant.

## Results

**Fig. 1:**
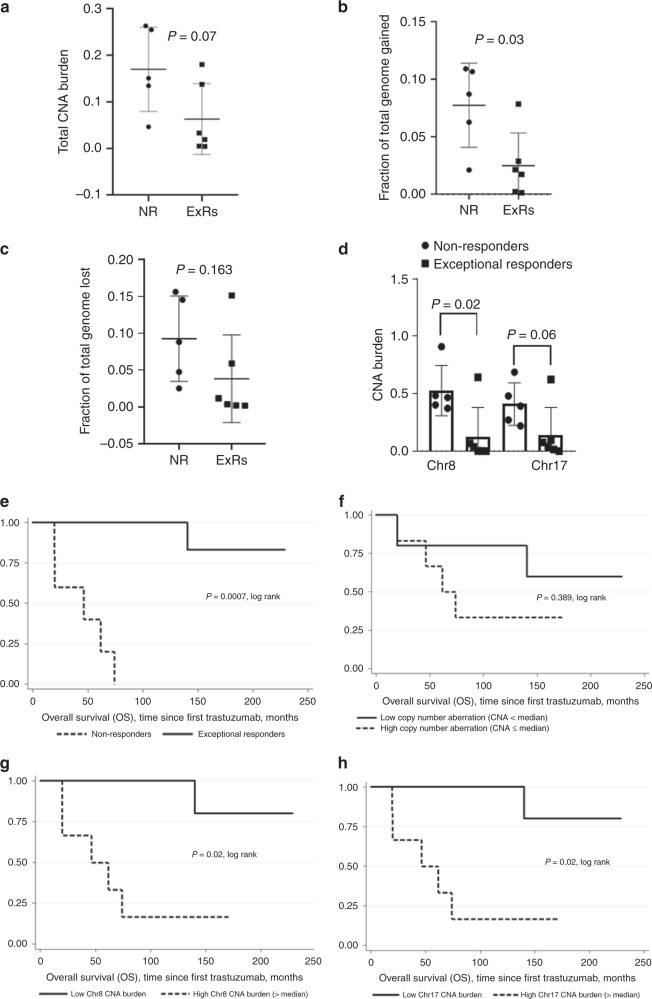
Results of CNA burden on ExRs and NRs and overall survival outcome. **a** Total overall genome-wide CNA burden. **b** Fraction of genome gained. **c** Fraction of genome lost. **d** Individual chromosome CNA burden in Chr8 and Chr17 in non-responder (NR) and exceptional responder (ExRs) genomes. **e** Kaplan–Meier overall survival curves of cases dichotomised by exceptional responder and non-responder. **f** Total CNA burden, low CNA (total CNA burden < median) and high CNA (total CNA ≥ median). **g** Overall CNA burden on Chr8, low CNA (Chr8 total CNA burden < median) and high CNA (Chr8 total CNA burden ≥ median). **h** Overall CNA burden on Chr17, low CNA (Chr17 total CNA burden < median) and high CNA (Chr17 total CNA burden ≥ median).

We estimated the fraction of the genome amplified/deleted and present CNA burden as a measure of genome instability. We observed that the overall fraction of genome CNA burden was higher in NR patients (*P* = 0.07; Fig. 1a), while more significantly pronounced in copy number gains (*P* = 0.03) in NRs compared to ExRs (Fig. 1b), with non-significant copy number losses (Fig. 1c). We further delineated the distribution of CNA burden across the genome and identified a greater degree of CNA burden in NRs in chromosome 8 (*P* = 0.02) and in chromosome 17 (Fig. 1d). Kaplan–Meier survival analysis highlighted the extended survival of ExRs compared to NRs (*P* = 0.0007, log rank; Fig. 1e). Further analysis highlighted that dichotomisation into low versus high CNA burden does not significantly affect overall survival (*P* = 0.389, log rank); however, it does stratify patients (Fig. 1f). Subanalysis of the CNA burden at specific chromosomes revealed that low total CNA burden at Chr8 and Chr17 conferred a statistically significant benefit in overall survival (*P* = 0.02 and *P* = 0.02, log rank; Fig. 1g, h).

To provide additional insight into the somatic mutation landscape at variant, gene, and biological pathway levels, we refined our study to include only normal–tumour matched pairs of ExR (*n* = 5; 3 never relapse, 2 RFS >96 months) and NR (*n* = 4) cases. Of our small cohort, we observed a higher median of SNV in ExRs (median = 1621) compared to NRs (median = 638); albeit this large variance was mostly driven by an unusual SNV in ExR patient 1 (Supplementary Fig. 1a, b). Mutations were observed in *TTN* (80% of ExR and 75% NR samples), *HSPG2* (60% of ExR and 75% NR samples), *SYNE1* (40% of ExR and 75% NR samples), *SYNE2* (80% of ExR and 50% NR samples) and *MACF1* (40% of ExR and 75% NR samples) (Supplementary Fig. 1c). We used the mutant-allele tumour heterogeneity (MATH) score to quantify intratumour heterogeneity based on the variation in variant’s allele frequencies of all mutations in a tumour. MATH scores were computed for the ExR and NR tumours separately. MATH scores from ExR (mean = 47, median = 41, interquartile range (IQR) = 16–46) and NR (mean = 35, median = 31, IQR = 23–102) and TMB was insignificantly different between both cohorts (Supplementary Fig. 1d, e).

## Discussion

Identification of genomic alterations associated with exceptional response and survival may improve risk assessment and treatment strategies for HER2+ MBCs. This is the first study to propose that CNA burden in HER2+ MBC ExRs may represent a novel prognostic predictor to trastuzumab response. Despite the limited sample size, we observed a trend in which the overall CNA burden was lower in ExRs compared to NRs; moreover, individual CNA analysis per chromosome revealed that specific chromosomes 8 and 17 were more altered in NR genomes compared to ExRs, and stratified analysis revealed significantly poorer overall survival. CNA burden was previously shown to be associated with overall survival and disease-specific survival in breast cancer, with chromosome 8 along with chromosomes 1 and 16 carrying the highest CNA burden, suggesting a further role of chromosome 8 in prognosis.^13^ Key genes in our analysis such as *TTN, HSPG2, SYNE1*, *SYNE2* and *MACF1* are frequently altered in breast and other cancers but their roles in HER2+ breast cancer tumorigenesis and trastuzumab response/resistance are as yet uncertain. Our investigation of genome-wide CNA burden offers the potential to gain insight into the underlying genetic landscape of long-term, never relapse exceptional response to trastuzumab. Recent analysis of the SAKK 22/99 trial identified that a subset of advanced HER2+ patients displayed long-term disease control with trastuzumab monotherapy;^14^ however, this study was unable to identify any meaningful clinical predictive markers to characterise these patients. Therefore, our preliminary study supports our hypothesis that CNA burden may account for exceptional response to trastuzumab. Particularly as MBC is generally termed incurable, this study presents a paradigm shift in the conventional ideology of oncology therapeutics that is unexplored and clearly warrants further investigation. The contribution of the immune system to the therapeutic effect of trastuzumab and other HER2 antibodies has been established, and further investigation into immune-related markers may provide predictive information for increased clinical activity in combination with genomic CNA data. Therefore, an extended analysis of 40 HER2+ MBC ExRs and NRs is now underway to validate our findings; furthermore, we intend to characterise the role of CNAs and immune signatures that may contribute to long-term trastuzumab (and other HER2 therapies) therapeutic response in the metastatic setting.

## Supplementary information

Supplementary files

## Data Availability

Whole-exome sequencing and clinical data from this project is available from EGA (accession number: EGAS00001004486). Archive for academic research within the constraints of the consent given from patients. All bioinformatics tools are fully described in “Methods”.

## References

[1] Slamon DJ, Leyland-Jones B, Shak S, Fuchs H, Paton V, Bajamonde A (2001). Use of chemotherapy plus a monoclonal antibody against HER2 for metastatic breast cancer that overexpresses HER2. N. Engl. J. Med..

[2] Swain SM, Baselga J, Kim SB, Ro J, Semiglazov V, Campone M, Ciruelos E (2015). Pertuzumab, trastuzumab, and docetaxel in HER2-positive metastatic breast cancer. N. Engl. J. Med..

[3] Gullo G, Zuradelli M, Sclafani F, Santoro A, Crown J (2012). Durable complete response following chemotherapy and trastuzumab for metastatic HER2-positive breast cancer. Ann. Oncol..

[4] Gámez-Pozo A, Pérez Carrión RM, Manso L, Crespo C, Mendiola C, López-Vacas R (2014). The Long-HER Study: clinical and molecular analysis of patients with HER2+ advanced breast cancer who become long-term survivors with trastuzumab-based therapy. PLoS ONE.

[5] Pereira B, Chin SF, Rueda OM, Moen Vollan HK, Provenzano E, Bardwell HA (2016). The somatic mutation profiles of 2,433 breast cancers refines their genomic and transcriptomic landscapes. Nat. Commun..

[6] Ciriello G, Miller ML, Aksoy BA, Senbabaoglu Y, Schultz N, Sander C (2013). Emerging landscape of oncogenic signatures across human cancers. Nat. Genet..

[7] Li H, Durbin R (2010). Fast and accurate long-read alignment with Burrows-Wheeler transform. Bioinformatics.

[8] Depristo MA, Banks E, Poplin R, Garimella KV, Maguire JR, Hartl C (2011). A framework for variation discovery and genotyping using next-generation DNA sequencing data. Nat. Genet..

[9] D’Aurizio R, Pippucci T, Tattini L, Giusti B, Pellegrini M, Magi A (2016). Enhanced copy number variants detection from whole-exome sequencing data using EXCAVATOR2. Nucleic Acids Res..

[10] Mayakonda A, Lin DC, Assenov Y, Plass C, Koeffler HP (2018). Maftools: efficient and comprehensive analysis of somatic variants in cancer. Genome Res..

[11] Mroz EA, Rocco JW (2013). MATH, a novel measure of intratumor genetic heterogeneity, is high in poor-outcome classes of head and neck squamous cell carcinoma. Oral. Oncol..

[12] Yarchoan M, Hopkins A, Jaffee EM (2017). Tumor mutational burden and response rate to PD-1 inhibition. N. Engl. J. Med..

[13] Zhang L, Feizi N, Chi C, Hu P (2018). Association analysis of somatic copy number alteration burden with breast cancer survival. Front. Genet..

[14] Schmid S, Klingbiel D, Aebi S, Goldhirsch A, Mamot C, Munzone E (2019). Long-term responders to trastuzumab monotherapy in first-line HER-2+ advanced breast cancer: characteristics and survival data. BMC Cancer.

