# Supplementary material for: Whole-exome sequencing of long-term, never relapse exceptional responders of trastuzumab-treated HER2+ metastatic breast cancer
Source: Br J Cancer. 2020 Jul 27;123(8):1219–22. doi: 10.1038/s41416-020-0999-z (PMC7553955; doi:10.1038/s41416-020-0999-z)
Supplement: Supplementary file 1 — Supplementary files [file 41416_2020_999_MOESM1_ESM.docx]

**Supplementary Table 1. Summary of histopathological characteristics between long term, never relapse exceptional responders (ExRs) to trastuzumab and non-responders (NR)**

| **Patient characteristics** | **ExRs**  **N=6** | **NR**  **N=5** |
| --- | --- | --- |
| **Age (median, yrs) 1^st^ Trastuzumab for advanced disease**  Range (mo) | 54  31-78 | 50  39-62 |
| **Histology**  IDC  ILC | 5  1 | 5  0 |
| **Grade**  3  2 | 5  1 | 4  1 |
| **Hormone Receptors Estrogen/Progesterone**  Positive  Negative | 3  3 | 3  2 |
| **De novo stage IV**  Yes  No | 3  3 | 1  4 |
| **Number of metastatic organs**  1  2 | 5  1 | 1  4 |
| **Location of mets**  Lymph  Liver  Lung  Bone | 5  1  0  0 | 1  1  4  2 |
| **Clinical response**  **Complete response (CR)**  **Partial response (PR)**  **Unknown** | 5  1  0 | 0  4  1 |
| **Relapse-Free Survival Time (median, mo)**  1^st^ Trastuzumab for advanced disease to follow-up/progression  Range (mo) | n/a  n/a | 14  8-21 |
| **Overall Survival Time (median, mo)**  1^st^ Trastuzumab for advanced disease to follow-up/death  Range (mo) | 167  140-229 | 46  19-74 |


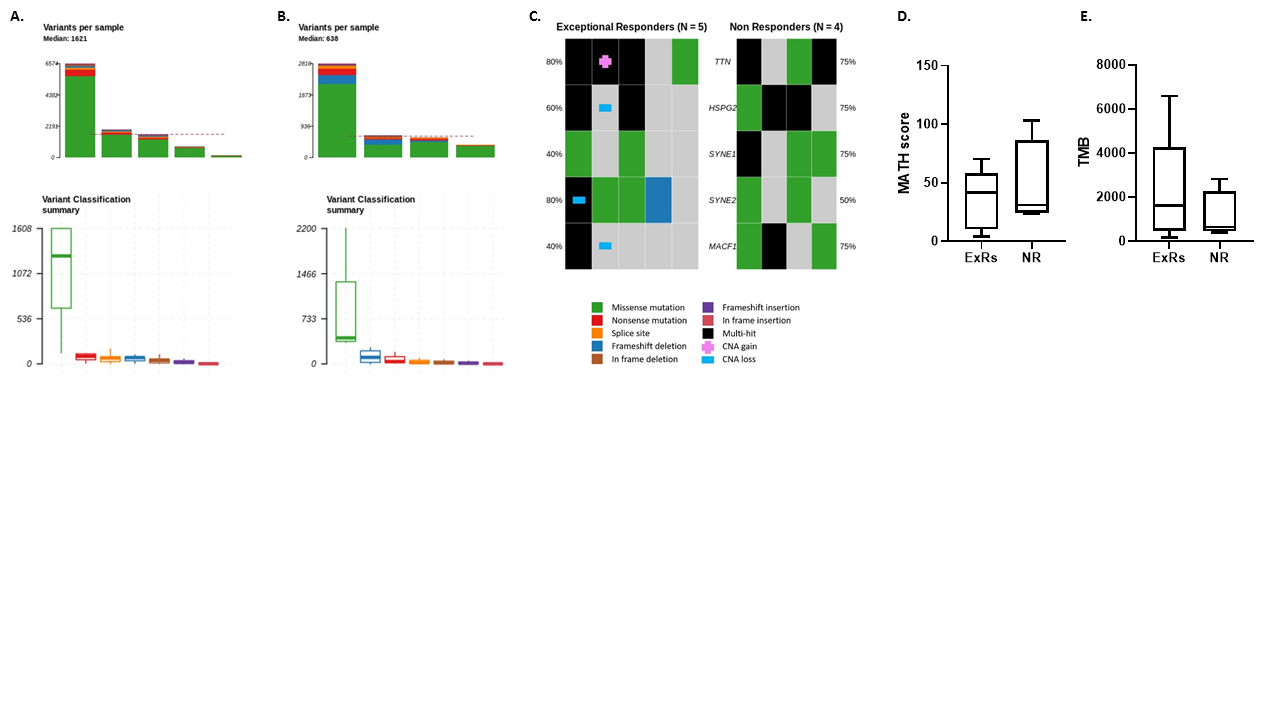


**Supplementary Figure 1. A. Variant statistic for Exceptional Responders B. Variant statistic for Non Responders samples C. Oncoplot of 5 most altered genes in cohort somatic genomes D. MATH score E. Tumor Mutation Burden (TMB) in Exceptional Responders and Non Responders.**
